# Focus on seed cells: stem cells in 3D bioprinting of corneal grafts

**DOI:** 10.3389/fbioe.2024.1423864

**Published:** 2024-07-10

**Authors:** Zi-jun Xie, Bo-wei Yuan, Miao-miao Chi, Jing Hong

**Affiliations:** ^1^ Department of Ophthalmology, Peking University Third Hospital, Beijing, China; ^2^ Beijing Key Laboratory of Restoration of Damaged Ocular Nerve, Peking University Third Hospital, Beijing, China

**Keywords:** cornea, 3D bioprinting, stem cells, regenerative medicine, keratoplasty

## Abstract

Corneal opacity is one of the leading causes of severe vision impairment. Corneal transplantation is the dominant therapy for irreversible corneal blindness. However, there is a worldwide shortage of donor grafts and consequently an urgent demand for alternatives. Three-dimensional (3D) bioprinting is an innovative additive manufacturing technology for high-resolution distribution of bioink to construct human tissues. The technology has shown great promise in the field of bone, cartilage and skin tissue construction. 3D bioprinting allows precise structural construction and functional cell printing, which makes it possible to print personalized full-thickness or lamellar corneal layers. Seed cells play an important role in producing corneal biological functions. And stem cells are potential seed cells for corneal tissue construction. In this review, the basic anatomy and physiology of the natural human cornea and the grafts for keratoplasties are introduced. Then, the applications of 3D bioprinting techniques and bioinks for corneal tissue construction and their interaction with seed cells are reviewed, and both the application and promising future of stem cells in corneal tissue engineering is discussed. Finally, the development trends requirements and challenges of using stem cells as seed cells in corneal graft construction are summarized, and future development directions are suggested.

## 1 Introduction

Corneal opacity is the fourth leading cause of severe vision impairment worldwide (World Health Organization, 2019). The opacity may be caused by trauma, infections, congenital diseases, etc. As a leading cause of irreversible vision impairment in all age groups, corneal diseases seriously affect quality of life and have a high social burden (Pascolini and Mariotti, 2012; Mathews et al., 2018). Corneal transplantation, also known as keratoplasty, is the main treatment for corneal opacity (Tan DTH. et al., 2012; Singh et al., 2019). However, there is a severe shortage of corneal graft tissue relative to its demand. According to a study from 2016, only one cornea is available for every 70 that are needed, and approximately 53% of the global population has no opportunity for corneal transplantation, leading to inevitable corneal blindness for a large population of patients with severe corneal disease (Gain et al., 2016).

To mitigate the shortage of donor tissue, alternatives for donor grafts should be urgently developed in addition to encouraging cornea donation. Keratoprosthesis has been used as a solution in clinical practice since the mid-20th century (Zhang et al., 2019a). However, postoperative complications, including the development of retroprosthetic membrane, glaucoma, retinal detachment, infectious endophthalmitis and choroidal detachment, may occur due to the material of corneal prosthesis and the complex surgical techniques (Wang et al., 2023). Among these complications, retroprosthetic membrane due to immunological reaction to the foreign device is the most common complication (Tan A. et al., 2012; Lee et al., 2017; Saeed et al., 2017; Park et al., 2020). In contrast to keratoprosthesis, tissue engineered corneal grafts may be a better solution (Tan DTH. et al., 2012; Wilson et al., 2013; Wang et al., 2023).

Three-dimensional (3D) bioprinting has been widely used in tissue engineering of cartilage, bone and skin (Murphy et al., 2020). It has the advantages of living cell printing, multilayer printing, composite material printing, high-resolution printing and automated production (Shiju et al., 2020). However, product integration into the host vascular network remains a difficult problem (Murphy et al., 2020). In addition, the presence of allogeneic cells in the product may cause immune rejection. The cornea is a tissue without blood supply, which makes the cornea a tissue with immune privilege. Therefore, 3D printing has promising potential in the construction of corneal tissue.

However, 3D printing technology has a stimulating effect on cells and can decrease cell viability (Xu et al., 2022). In addition, enough seed cells must be added to the bioink to ensure the biological function of the product. However, the essential cellular components of corneal tissue, including stromal cells and endothelial cells, etc., are difficult to expand *in vitro* (Yam et al., 2015a; Hazra et al., 2022). Therefore, it is highly important to find suitable seed cells to replace the primary cells for corneal tissue engineering. Stem cells have a strong ability to proliferate and differentiate. Great breakthroughs have been made in the research on differentiation induction strategies and the cell function of stem cells recently, which provides possibilities for their application in 3D bioprinting.

Research to explore the 3D bioprinting of corneal tissue has been conducted since 2016. This review summarizes the anatomical and physiological characteristics of the cornea. The applications of 3D bioprinting in corneal tissue are then reviewed. The prospects and applications of stem cells as promising seed cells are discussed. Finally, perspectives on the use of stem cells in 3D bioprinting of corneal graft tissue are introduced.

## 2 Cornea: anatomy and physiology

The cornea is part of the outer shell of the eye, protecting the inner components from physical injury and maintaining the shape of the eyeball. As the window of the eye, the cornea allows the entry of light and helps focus that light on the retina through its transparency, surface smoothness, geometrical shape and refractive index.

**FIGURE 1:**
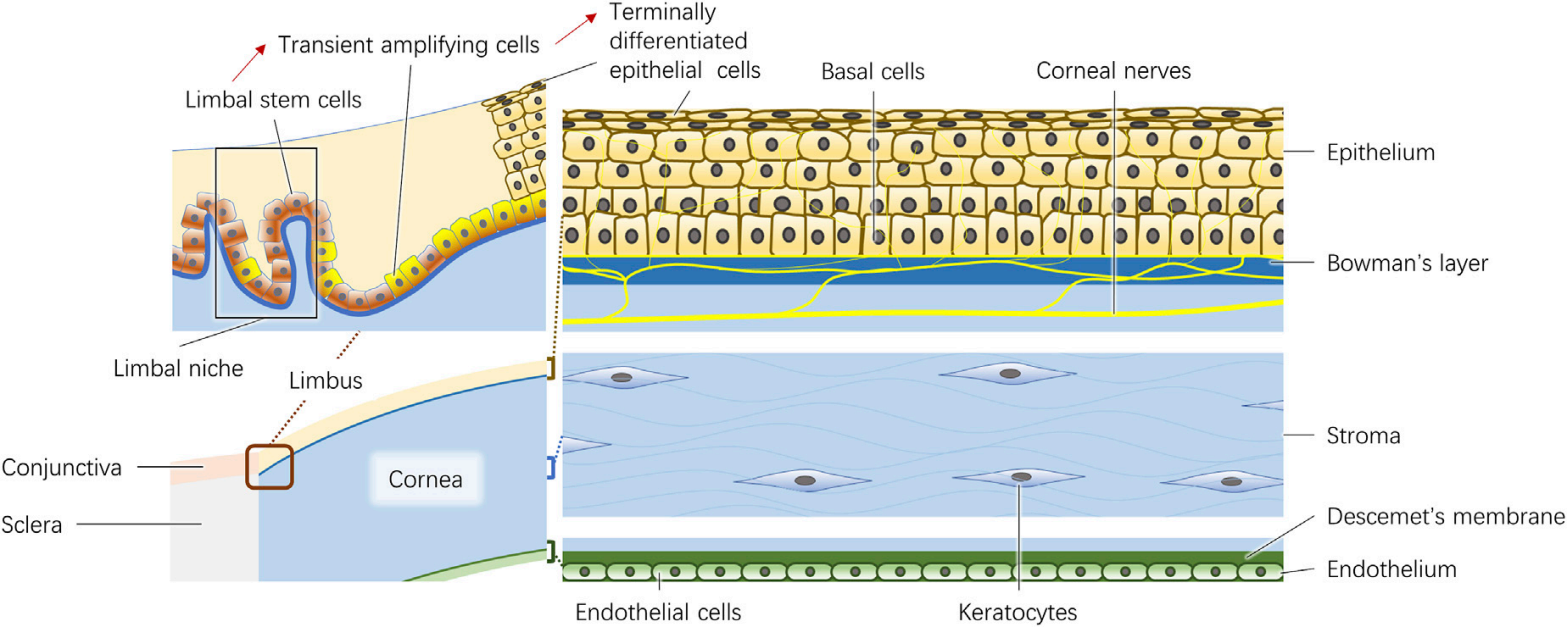
Schematic diagram of the limbus and the five layers of cornea.

The adult human cornea is approximately 500 μm thick at the center. It consists of the epithelium, Bowman’s layer, stroma, Descemet membrane and endothelium (Figure 1). In addition, the corneal nerves play a significant role in the trophic function of corneal cells.

### 2.1 Epithelium

The epithelium is approximately 50 μm thick and is composed of five to six layers of superficial cells, wing cells and columnar basal cells. The junctional complexes between epithelial cells create a barrier as a biodefense system on the ocular surface (Leong and Tong, 2015). Both the basal cells and the limbal stem cells, which are believed to be in the palisades of Vogt at the limbus, are involved in the dynamically balanced renewal process of the corneal epithelium (Li et al., 2007). The limbal epithelial stem cells in the limbal niche produce transient amplifying cells, and finally differentiate into terminal differentiated epithelial cells (Figure 1) (Yazdanpanah et al., 2017). Thoft and Friend proposed a classical equilibrium hypothesis represented by equation X + Y = Z in 1983 (Thoft and Friend, 1983). This hypothesis states that the combination of the proliferation and differentiation of basal cells (X) and the centripetal movement of peripheral epithelial cells differentiated from limbal stem cells (Y) is equivalent to epithelial loss from the corneal surface (Z), highlighting the importance of basal cells and limbal stem cells in the maintenance of epithelial integrity (Thoft and Friend, 1983). In addition, the basement membrane to which basal cells attach plays an important role in the formation of cell‒cell junctions during reformation of the corneal epithelium (Suzuki et al., 2000).

### 2.2 Bowman’s layer and stroma

Bowman’s layer is a 10 μm thick arrangement of collaged fibrils and proteoglycans (Germundsson et al., 2013). Considering its shadowy functions, it is usually regarded as the anterior constituent of the corneal stroma (Wilson, 2023).

The stroma constitutes the majority of the cornea, making up 90% of the thickness of the entire cornea. The anatomic and physiological properties of the stroma, including its physical strength, morphological stability, refractivity and transparency, strongly contribute to the characteristics of the cornea. The extracellular matrix (ECM) constitutes the largest portion of the stroma and is mostly composed of collagen (types I and V) and lesser amounts of proteoglycans (decorin, lumican, keratocan and mimecan) (Birk et al., 1986; Hassell and Birk, 2010). The collagen fibrils, which are 25∼30 nm in diameter, are distributed homogeneously and packed regularly in lamellae, and adjacent layers form an orthogonal lattice, allowing entrance of the light (Hassell and Birk, 2010). An adult cornea is composed of 300 collagen lamellae in the central region and 500 close to the limbus (Radner et al., 1998). 3D analysis revealed that the angle between the lamellae is altered from Bowman’s layer to Descemet’s membrane, which increases the difficulty of simulating biological corneal structures with 3D bioprinting (Morishige et al., 2014). As a matter of course, the cornea loses its transparency once the alignment and thickness of the fibrils become heterogeneous (Hassell and Birk, 2010). Although they comprise only a small fraction of the stroma, cellular components, which include keratocytes and bone marrow-derived cells, are fundamental in stroma transparency maintenance and tissue repair. As the resident cells of the stroma, they connect with each other by extending their long processes and usually remain quiescent in normal corneas (Ueda et al., 1987). Once activated by any insult, the keratocytes transform into myofibroblasts or fibroblasts and secrete ECM, enzymes, mechanistic metalloproteinases and cytokines to repair stromal injury (Yam et al., 2020).

### 2.3 Descemet’s membrane and endothelium

Descemet’s membrane is the basement membrane of corneal endothelial cells. The membrane is composed of laminin, heparan sulfate proteoglycans, fibronectin, nidogens and collagen types IV and VIII (Kefalides and Denduchis, 1969; Labermeier and Kenney, 1983; Leung et al., 2000; Kabosova et al., 2007; Medeiros et al., 2018).

The corneal endothelium is a single layer of corneal endothelial cells arranged in a mosaic pattern. Healthy endothelial cells are usually hexagonal, and they produce the typical pattern of collagen VIII. They contain various junctional complexes and ion transport systems to prevent fluid from entering the stroma. Usually, endothelial cells are arrested in G1 phase in human eyes, so the loss of endothelial cells leads to stromal edema and loss of corneal transparency (Joyce et al., 1996; Price et al., 2021). Considering the nonproliferative nature of endothelial cells *in vivo*, *in vitro* expansion seems to be an alternative method to obtain sufficient amounts of living endothelial cells (Liu et al., 2017). However, endothelial cells may undergo endothelial-mesenchymal transition (EMT), leading to phenotypic and functional changes, increasing the difficulty of obtaining seed cells (Roy et al., 2015). Recently, the *in vitro* cell culture by the dual media enriched by additional substances including EMT inhibitors was developed (Smeringaiova et al., 2021). Repeated alternation of high and low mitogenic conditions were carried out for dual cell culture. This technique was applied in the first in-human clinical trial using cultured endothelial grafts (Kinoshita et al., 2018). However, the long-term effect remains to be researched.

## 3 Keratoplasty: from full-thickness to lamellar graft

**FIGURE 2:**
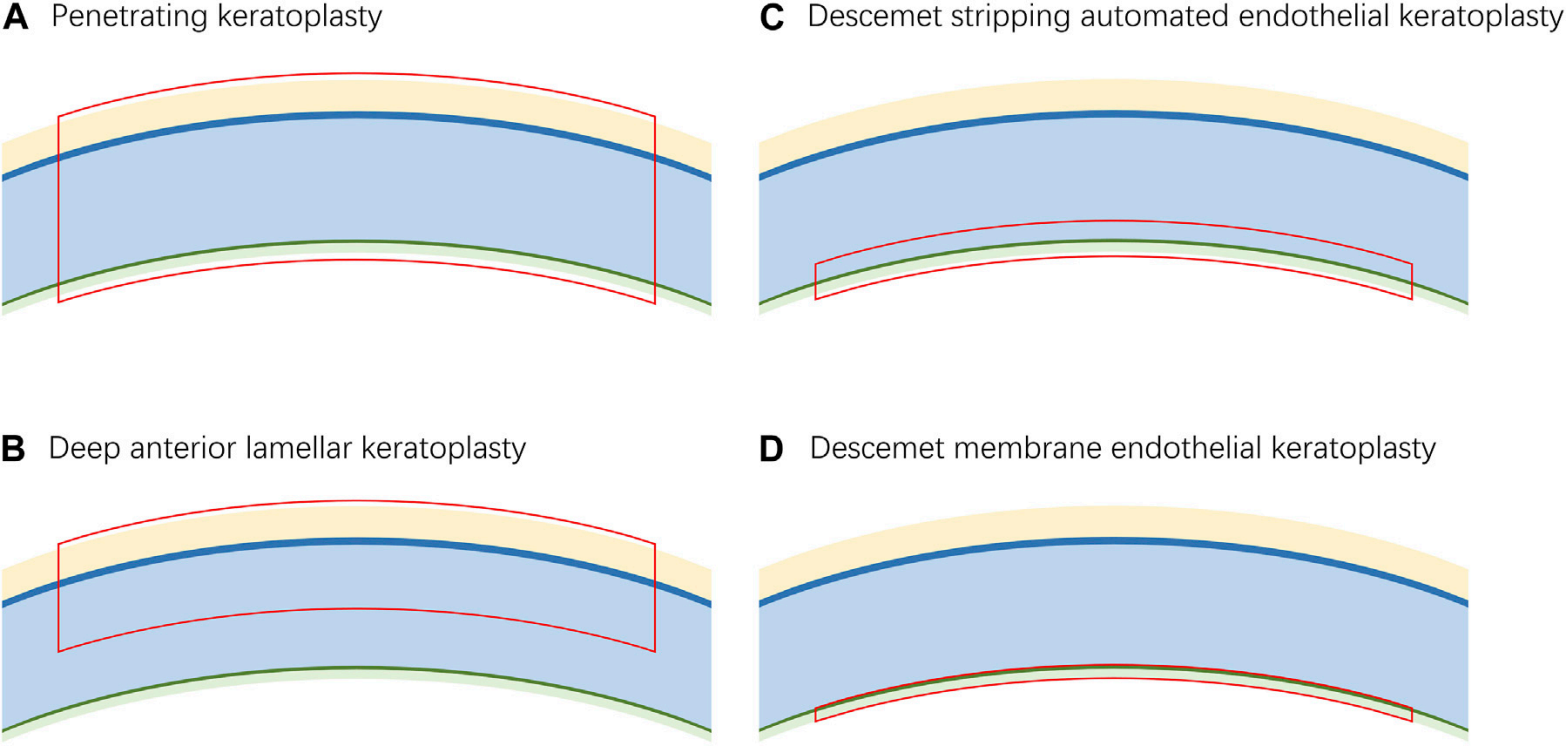
The corresponding graft layers of different keratoplasties. **(A)**. The graft for penetrating keratoplasty requires a full-thickness cornea. **(B)**. The graft for deep anterior lamellar keratoplasty requires the epithelium, Bowman’s layer and stroma. **(C)**. The graft for Descemet stripping automated endothelial keratoplasty requires a fraction of the stroma, Descemet’s membrane and endothelium. **(D)**. The graft for Descemet membrane endothelial reconstruction requires Descemet’s membrane and endothelium.

Keratoplasty is the most successful allogenic transplant worldwide (Singh et al., 2019). In 1906, Eduard Zirm successfully performed the first keratoplasty (Zirm, 1989). Since then, keratoplasty has developed rapidly. However, the implementation of classical keratoplasty, currently known as penetrating keratoplasty (PKP), is dependent on the availability of full thickness donor material, which is usually in short supply (Figure 2A) (Gain et al., 2016). In the late 19th century, the concept of lamellar keratoplasty was proposed and developed (Mannis and Holland, 2017). This new method not only minimizes the use of donor materials but also reduces the complications of keratoplasty (Akanda et al., 2015; Hos et al., 2019). Lamellar keratoplasty entails the selective removal of the lesion layers and retention of the normal tissue, which creates a need for corneal grafts containing different layers.

The most commonly used lamellar keratoplasty procedures include deep anterior lamellar keratoplasty (DALK), Descemet stripping automated endothelial keratoplasty (DSAEK) and Descemet membrane endothelial keratoplasty (DMEK). DALK was first successfully performed by Malbran in 1972 (Malbran and Stefani, 1972). Currently, it has become one of the most preferred surgeries for the treatment of corneal stromal disease (Thanitcul et al., 2021). The grafts used in DALK consist of the epithelium, Bowman’s layer and part of the stroma of the donor material (Figure 2B).

Corneal endothelial disease is the most common reason for corneal transplantation in industrialized countries (Tan DTH. et al., 2012). DSAEK and DMEK were both proposed to target corneal endothelial disease in 2006 (Gorovoy, 2006; Melles et al., 2006). The grafts used in DSAEK contain the endothelium, Descemet’s membrane and a fraction of stroma, while the grafts used in DMEK contain only the endothelium and Descemet’s membrane, which allows more accurate anatomical reconstruction of the diseased cornea (Figures 2C, 2D). Studies have shown that eyes that have undergone DMEK have better best corrected visual acuity than those that have undergone DSAEK; however, the learning curve of DMEK is relatively difficult, which has limited its popularization (Dapena et al., 2009; Terry, 2012; Stuart et al., 2018; Stuhlmacher et al., 2023). For example, the risk of preparation failure and low tissue quality in manual preparation of the graft are challenges (Terry, 2012; Birbal et al., 2018). However, 3D bioprinting may be a promising technique for automatic preparation procedures.

In view of the current shortage of corneal donors, alternative methods must urgently be explored. As a technique that can be used for the fabrication of multilayer composite biomaterials containing cells, 3D bioprinting holds great potential to construct various corneal grafts.

## 4 Overview of 3D bioprinting techniques used in cornea construction

3D bioprinting is an additive manufacturing (Gungor-Ozkerim et al., 2018). Complex human tissues can be constructed using this method by precisely spatially distributing bioinks containing living cells (Mandrycky et al., 2016).

### 4.1 Extrusion-based bioprinting

**FIGURE 3:**
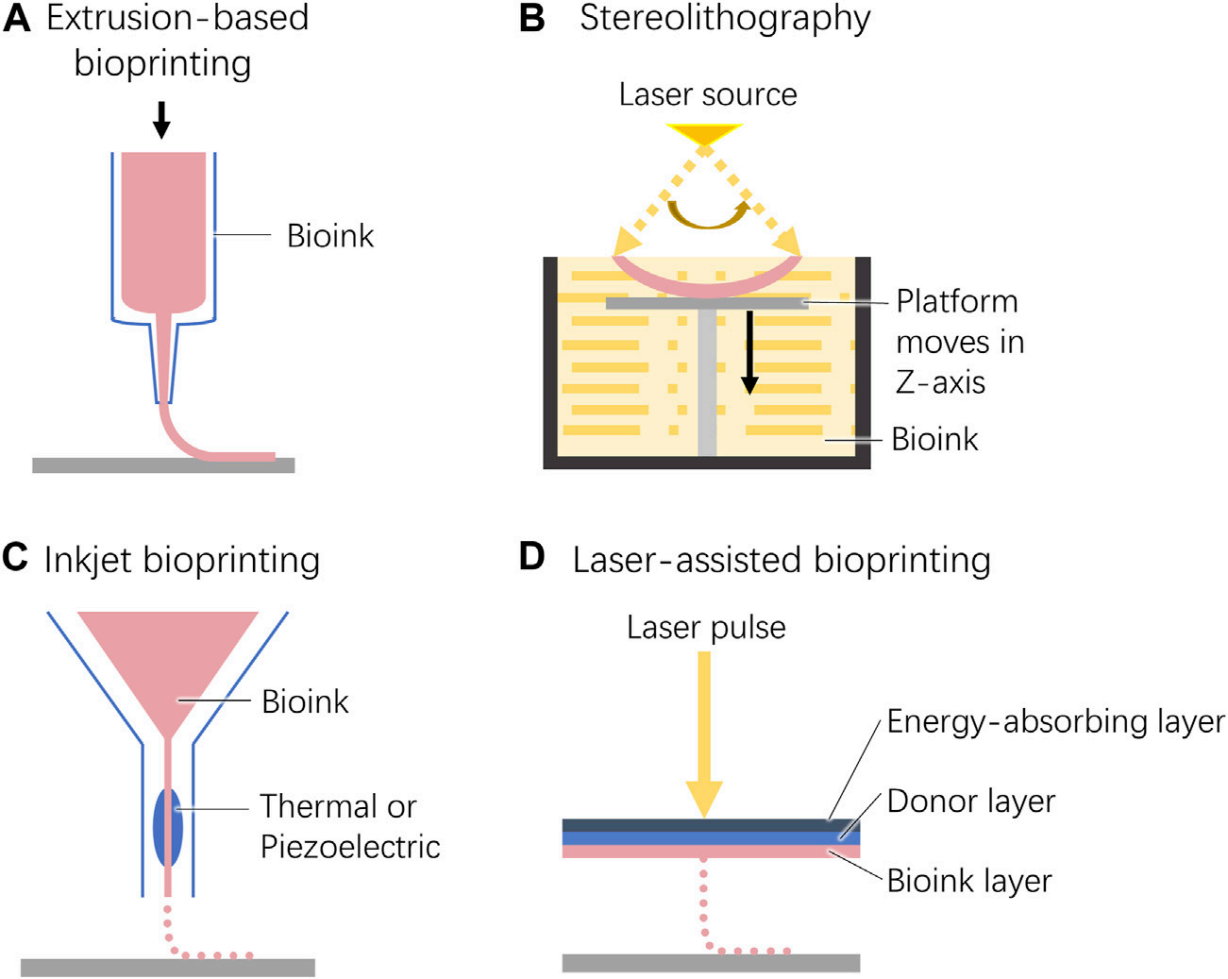
Schematic diagram of the 3D bioprinting techniques used for cornea construction. **(A)**. Extrusion-based bioprinting. **(B)**. Stereolithography. **(C)**. Inkjet bioprinting. **(D)**. Laser-assisted bioprinting.

Extrusion-based bioprinting is the first 3D bioprinting strategy applied to corneal tissue construction (Figure 3A) (Zein et al., 2002; Wu et al., 2016). Both pneumatic- and mechanical-based drive systems have been used to implement this method. There are multiple types of pneumatic-based systems including both valve-based and valve-free systems. The former is commonly used for bioprinting due to its simple operation. The printing accuracy can be adjusted by installing nozzles of different sizes (Ozbolat and Hospodiuk, 2016). In contrast, the latter enables high-precision resolution printing with pressure and pulse control (Mandrycky et al., 2016).

### 4.2 Stereolithography

During stereolithography, liquid bioinks are selectively solidified by layer-by-layer photocrosslinking to generate complex 3D structures (Figure 3B) (Heinrich et al., 2019). This technology requires high transparency of the bioink to ensure that light can pass through the material and produce uniform cross-linking. Therefore, the cell density within the bioink is limited to 10^8^ cells/mL (Mandrycky et al., 2016).

### 4.3 Inkjet bioprinting

Similar to traditional 2D inkjet printing, 3D inkjet printing constructs a three-dimensional structure by building layers along the *z*-axis of the system (Figure 3C) (Singh et al., 2010; Heinrich et al., 2019).

### 4.4 Laser-assisted bioprinting

Laser-assisted bioprinting is derived from laser direct-write and laser-induced transfer technologies (Mandrycky et al., 2016). From top to bottom, the working platform consists of an energy-absorbing layer, a donor layer, and a bioink layer. When an exogenous laser beam is applied to a certain position of the energy absorption layer, a high-pressure bubble is generated at the corresponding position of the donor layer, which pushes the biological ink down to drop onto the construction platform (Figure 3D).

## 5 Seed cells

Seed cells play a crucial role in maintaining the biological functionality of 3D bioprinted products (Ma et al., 2024). However, an ideal cell source that fully meets the requirements of 3D bioprinting has not yet been identified due to the specific application characteristics of different sources. In this section, we review the applications of potential cell sources including cell lines, primary cells, stem cells, and transdifferentiated cells for 3D bioprinting.

### 5.1 Primary cells

Primary cells have been widely used for their excellent functionality and safety in bioprinting applications (Ma et al., 2024). However, the donor source, replicative senescence and *in vitro* culture cause special challenges (Ma et al., 2024). In the process of 3D printing, primary cells are more susceptible to damage caused by the printing process (Ma et al., 2024). In addition, the clinical use of primary cells is limited by ethical considerations of resource allocation brought by the limited source.

The *in vitro* protocols for epithelial cell culture are well established (Castro-Muñozledo, 2008). However, the corneal epithelial layer is not necessary for the construction of grafts in keratoplasty for patients with healthy limbal corneal stem cells.

In contrast, corneal stromal cells and endothelial cells may easily lose their original phenotypes during *in vitro* culture (Yam et al., 2015a; Hazra et al., 2022). Corneal stromal keratocytes can transform into fibroblasts, proliferate and migrate during *in vitro* culture, and upregulate the expression of ECM proteins, leading to graft opacification (Yam et al., 2015a; Yam et al., 2020). However, corneal endothelial cells usually undergo endothelial-mesenchymal transformation *in vitro* culture and acquire the phenotype of fibroblasts, resulting in loss of their original morphology, pump and barrier functions (Yamashita et al., 2018a). Therefore, due to the limitations of donor sources and the difficulty of *in vitro* expansion, primary cells are inadequate for large-scale 3D bioprinting of corneal tissue.

### 5.2 Cell lines

Cell lines have a stable phenotype and function, a relatively mature culture protocol, and strong resistance to stress and environmental changes (Clark et al., 2022; Ma et al., 2024). However, they have a worrisome capacity of expansion and genetic mutations, which increase the risk of tumor formation (Wu et al., 2022). Moreover, cell lines do not correspond perfectly to the specialized functions of primary cells *in vivo* (Rubelowski et al., 2020). Therefore, it is necessary to find cell lines with controlled proliferation and mature phenotype for corneal tissue engineering (Ma et al., 2024).

### 5.3 Transdifferentiated cells

Guo and Cieślar-Pobuda successfully transdifferentiated skin epidermal stem cells and fibroblasts into corneal epithelioid cells (Cieślar-Pobuda et al., 2016; Guo et al., 2018). Several breakthroughs have been made in corneal cell transdifferentiation strategies, but their application in corneal tissue engineering remains to be explored.

### 5.4 Stem cells

Stem cells have extraordinary self-renewal capabilities and pluripotency. By adding stem cells to bioinks, the requirement for donor-derived cells may be avoided, which is the main factor limiting corneal tissue engineering (Wu et al., 2012). Compared to that of cell lines, their proliferation decreases gradually during differentiation, which increases the safety of regeneration therapies. Due to the obstacles caused by the *in vitro* culture of keratocytes and endothelial cells, pluripotent stem cells have shown great promise in the 3D bioprinting of corneal stromal and endothelial tissue (Yam et al., 2015a; Yam et al., 2020; Hazra et al., 2022). In addition, mesenchymal stem cells (MSCs) are widely used due to their unique immunomodulatory function, especially in the field of tissue transplantation (Ma et al., 2006; Joyce et al., 2012; Podestà et al., 2020). This section provides an overview of research advances in the use of stem cells as a source of corneal seed cells.

#### 5.4.1 Corneal epithelial cells derived from stem cells

Limbal epithelial stem cells are intrinsic stem cells that reside on the corneal limbus, and are widely used for epithelial regeneration (Pellegrini et al., 1997; Kumar et al., 2022). Difficult ocular surface diseases have been successfully managed by transplantation of bioengineered tissue seeded with cultured corneal epithelial stem cells (Schwab et al., 2000). However, great progress has been made in the derivation of corneal epithelial cells from pluripotent stem cells (PSCs) and MSCs.

Moreover, pluripotent stem cells have the potential for multilineage differentiation. By blocking the TGF-β/Nodal and Wnt/β-catenin signaling pathways and adding bFGF, human PSCs were successfully induced to differentiate into corneal epithelial lineage (Hayashi et al., 2012; Mikhailova et al., 2014; Hayashi et al., 2017; Hongisto et al., 2018). Currently, differentiation-inducing methods based on xeno-free basal medium are proposed and optimized (Yang et al., 2018; Abdalkader and Kamei, 2022). Mechanical isolation and induction of the surface ectoderm of multi-zone ocular cells for epithelial cells derivation also explored a new direction for the source of seed cells (Li et al., 2019).

For MSCs, Gu et al. (2009) first successfully derived cells with morphological characteristics of corneal epithelial cells from MSCs in addition to expressing the corneal epithelial cell marker CK3 in the presence of limbal stem cells (LSCs) supernatant. Thereafter, corneal epithelial cells were derived by strategies such as coculture with corneal stromal cells, IGF-I induction, and culture on fresh pig corneal Bowman’s membrane (Hou et al., 2010; Jiang et al., 2010; Trosan et al., 2012; Park et al., 2021).

In particular, Galindo et al. (2017) reported that when inoculated on the cornea, MSCs could acquire corneal epithelial-like phenotypes. Besides, it was found that the epithelial-mesenchymal transition in human corneal epithelium was attenuated by secretomes of adipose-derived MSCs (Shibata et al., 2019). These evidences suggest the guaranteed advantages of using MSCs directly or in combination for corneal tissue bioprinting.

#### 5.4.2 Corneal stromal keratocytes derived from stem cells

A variety of MSCs can differentiate into corneal stromal keratocytes, including adipose tissue-derived mesenchymal stromal cells (ASCs), bone marrow mesenchymal stem cells (BMSCs), induced pluripotent stem cells (iPSCs), periodontal ligament stem cells and especially corneal stromal stem cells (CSSCs), the intrinsic stem cells of the cornea (Du et al., 2010; Liu et al., 2012; Basu et al., 2014; Yam et al., 2015b; Naylor et al., 2016). ASCs can be easily obtained from low-invasive liposuction and derived into functional keratocytes both *in vitro* and *in vivo* with significant differentiation potential and do not induce immune or inflammatory responses (Venkatakrishnan et al., 2022). Thus, ASCs are widely used in corneal bioprinting technology.

This induction was mainly achieved by the addition of keratocytes differentiation medium supplemented with FGF-2 and TGF-β3 (Wu et al., 2013). In addition, the arrangement of ECM components and other topographical cues have also been proposed to regulate cell differentiation, suggesting the great potential of 3D bioprinting or even 4D bioprinting of corneal stroma tissues using MSCs as seed cells (Wu et al., 2012; Petroll et al., 2020). Numerous clinical studies have been conducted to evaluate the safety and efficacy of CSSCs, ASCs and BMSCs in corneal regeneration (Zhang et al., 2022). However, MSCs have the potential to differentiate into multiple cell types, making it challenging to ensure specific differentiation into keratocytes.

A study observing the differences in the behavior of different MSCs in 3D bioprinted corneal stroma tissue showed that both ASCSs, BMSCs and CSSCs had high proliferation activity in the product, while the metabolic activity of BMSCs and CSSCs was significantly increased (Boix-Lemonche et al., 2023). All MSC subtypes were able to produce and reconstitute collagen in the scaffold during the observation period, whereas CSSCs secreted significant amounts of pigment epithelium-derived factors at the same time, which are considered to have anti-inflammatory and antiangiogenic properties (Boix-Lemonche et al., 2023). Therefore, CSSCs seem to be more suitable for 3D bioprinting of corneal stroma (Boix-Lemonche et al., 2023). However, the study had several limitations. The alginate used in the study was not biocompatible, so all three types of MSCs failed to differentiate and retained their MSC phenotype (Boix-Lemonche et al., 2023). Therefore, it is necessary to observe the different behaviors of MSCs in different biomaterials to find the most suitable seed cells (Boix-Lemonche et al., 2023).

#### 5.4.3 Corneal endothelial cells derived from stem cells

ASCs, ESCs, iPSCs, umbilical cord-derived mesenchymal stem cells (UC-MSCs) and PSCs were found to be potential seed cells in recent studies (Dai et al., 2014; Zhang et al., 2014; McCabe et al., 2015; Song et al., 2016; Zhao and Afshari, 2016; Chen et al., 2018; Wagoner et al., 2018; Yamashita et al., 2018b; Li et al., 2019; Chen et al., 2021; Gong et al., 2021; Grönroos et al., 2021; Marta et al., 2021; Sun et al., 2021; So et al., 2022; Ye et al., 2022). Moreover, human bone marrow-derived endothelial progenitor cells (BEPCs), skin-derived precursors (SKPs), cornea-derived precursors (COPs), rabbit oral mucosa epithelial cells and rat neural crest cells (rNCCs) have also been successfully derived from corneal endothelial cell-like cells (Shao et al., 2011; Ju et al., 2012; Hatou et al., 2013; Jiang et al., 2014; Inagaki et al., 2017; Shen et al., 2017).

Major derivation strategies included coculture with conditioned medium from corneal stromal cells and lens epithelial cells, the addition of all-trans retinoic acid and lens epithelial cell-conditioned medium, dual Smad inhibition, Wnt inhibition/activation, and ROCK inhibition (Dai et al., 2014; McCabe et al., 2015; Song et al., 2016; Zhao and Afshari, 2016; Chen et al., 2018; Wagoner et al., 2018; Yamashita et al., 2018b; Hatou and Shimmura, 2019; Li et al., 2019; Chen et al., 2021; Gong et al., 2021; Grönroos et al., 2021; Marta et al., 2021; Sun et al., 2021; So et al., 2022; Ye et al., 2022) (Table 1). However, cell behavior is affected by ECM stiffness. Therefore, to maintain the phenotype of induced corneal endothelial cell-like cells, the stiffness of 3D bioprinted products must be considered (Ali et al., 2016; Chaudhuri et al., 2020; Thomasy et al., 2024). Stem cell-derived endothelial cells have not been applied to corneal 3D bioprinting, which is definitely worth trying in the future.

**TABLE 1:** Summary of original studies on corneal endothelial progenitors.

Corneal endothelial progenitors	Species	Differentiation induction conditions	References
PSCs	Human	PSC→EFSC: N2B27 priming medium (DMEM/F12, N2, B27, 0.2% BSA, 2 mM L-GlutaMAX, 0.1 mM MEM nonessential amino acids and 0.1 mM β-mercaptoethanol) supplemented with small molecule inhibitors (5 μM SB431542, 50 nM LDN193189 and 1 μM IWP2) EFSC→NCSC: Neural crest induction medium (DMEM/F12: Neurobasal 50:50; N2, B27, 0.3 mM 2-phospho-L-ascorbic acid and 3 μM CHIR 99021) NCSC→CEC: CEC induction medium (human endothelial-SFM, 5% FBS, 0.3 mM 2-phosphate ascorbic acid and 1% P/S) supplemented with small molecular inhibitors (1 μM SB431542 and 2.5 μM H-1125)	Zhao and Afshari (2016)
PSCs	Human	iPSC→MZOC: Eye field differentiation medium (DMEM/F12 and neuralbasal medium supplemented with 2 mM L-GlutaMAX, 0.1 mM MEM non-essential amino acids, 0.1 mM β-mercaptoethanol, and 1% N2) for 7 dyas and ocular cell differentiation medium (DMEM/F12 supplemented with 10% KSR, 2 mM L-GlutaMAX, 0.1 mM NEAA, and 0.1 mM β-mercaptoethanol) for 3–4 weeks MZOC→CEC: Corneal endothelial differentiation medium (defined keratinocyte serum free medium/DMEM/F12 (1:1), 0.5% DMSO, 1% insulin-transferrin-selenium, 1 nM Cholera toxin, 0.2% Primocin, 5% KSR, 2 ng/μL EGF, and 10 μM Y-27632)	Li et al. (2019)
PSCs	Human	PSC→NCC: Serum-free basal medium (KnockOut Dulbecco’s Modified Eagle Medium, 15% KSR, 2 mM GlutaMax-I, 0.1 mM 2-mercaptoethanol, 50 U/mL P/S, 1% NEAA) supplemented with molecular inhibitors (500 nM LDN193189, 10 μM SB431542, 3 μM CHIR99021) NCC→CEC: Basal medium supplemented with 10 μM SB431542, 4 µM CHIR99021 and 10 µM retinoic acid	Grönroos et al. (2021)
ESCs	Human	hESC→CEC: Dual Smad induction media (80% DMEM/F12, 20% knock out serum replacement, 1% NEAA, 1 mM L-glutamine, 0.1 mM b-mercaptoethanol, and 8 ng/mL FGF2)	McCabe et al. (2015)
ESCs	Human	H9 hESCs→NCC: N2 medium (DMEM/F12, N2 supplement 5 mL, 10 ng/mL bFGF, 1X NEAA, 2 mM GlutaMAX™, 100 units/mL P/S, 2 ng/mL Human Recombinant Insulin) supplemented with 500 ng/mL Noggin and 10 μM SB431542 NCC→CEC-like cells: 1:1 fresh CEC medium (DMEM/F12 and M199, 5% FBS, 1X NEAA, 2 mM GlutaMAX™, 100 units/mL P/S, 2 ng/mL human recombinant insulin, L-Ascorbic Acid, 10 ng/mL bFGF) and collected bovine culture medium	Song et al. (2016)
ESCs	Human	hESC→POMP: HCSC–conditioned medium (human corneal stromal cell–conditioned medium) POMP→CEC-like cells: A mixture of with LEC-conditioned medium AND HCEC-conditioned medium	Chen et al. (2018)
iPSCs	Human	iPSC→NCC: basal medium (DMEM/F12 medium, 1 × N2, 1 × B27, 0.1 mM MEM with NEAA, 0.1 mM 2-mercaptoethanol and 10 ng/mL bFGF) supplemented with 2 µM SB431542 and 2 µM DMH1 NCC→CEC: chemically defined medium supplemented with small-molecule compounds. 20 ng/mL EGF and 2 µM CHIR99021 were added 3 days later	Chen et al. (2021)
iPSCs	Human	iPSC→NCC: neural crest differentiation medium (DMEM/F12 medium, 2% BSA, 200 ng/mL human insulin-like growth factor 1, 10 ng/mL Heregulinβ-1, 8 ng/mL FGF2, 50 μg/mL sodium L-ascorbic acid salt, 1% insulin-transferrin-selenium solution, 1% MEM non-essential amino acids solution, 0.1 mM 2-mercaptoethanol, 2 mM L-GlutaMAX, 2.0 μM SB431542, 1.0 μM CHIR99021, 1.0 μM DMH1, and 15 ng/mL bone morphogenetic protein 4) NCC→CEC-like cells: NCCs differentiate into CEC-like cells within the first 7 days after transplant	Gong et al. (2021)
iPSCs	Human	iPSC→NCC: Differentiation basal medium (DMEM/F12, 2% bovine serum albumin, 2 mM GlutaMAX, 0.1 mM MEM non-essential amino acid solution, 1× trace elements A, B, and C, 0.1 mM 2-mercaptoethanol, 50 μg/mL sodium L-ascorbate, 10 μg/mL transferrin, 10 ng/mL recombinant human Heregulin β-1, 200 ng/mL recombinant human LONGR3 IGF-I, 8 ng/mL recombinant human FGF2, 0.2% primocin, 10 µM SB431542 and 3 µM CHIR99021) NCC→CEC: Differentiation basal media supplemented with 0.1×B27, 10 ng/mL recombinant human PDGF-BB and 10 ng/mL recombinant human DKK-2	Wagoner et al. (2018)
iPSCs	Human	iPSC→NCC: basic culture medium (80% Dulbecco’s modification of Eagle’s medium-F12+GlutaMAX™-1, 20% KSR, 1% NEAA and 0.1 mM β-mercaptoethanol) supplemented with StemPro™ neural supplement, 20 ng/mL bFGF, 20 ng/mL EGF. NCC→CEC: CEC induction medium (basic culture medium supplemented with 8 ng/mL bFGF, 0.1×B27 supplement, 10 ng/mL recombinant human platelet derived growth factor-BB, 10 ng/mL recombinant human Dickkopf-related protein 2, 1 μM SB431542 and 2.5 μM Y27632)	Sun et al. (2021)
iPSCs	Human	iPSC→NCC: neural crest induction E6 medium supplemented with 500 nM LDN19318, 100 nM BGJ398, and 10 μM fasudil NCC→CEC: human endothelial-SFM supplemented with 0.1% polyvinyl alcohol, 1% insulin-transferrin-selenium, 0.2 mg/mL CaCl_2_, 0.02 mg/mL 2-phosphate ascorbic acid, 1 μM SB431542, 2.5 μM ROCK inhibitor H-1152 and 10 μM Y27632	So et al. (2022)
ASCs	Human	ASC→NCC: ① DMEM containing 1% FBS, 1% penicillin/streptomycin supplemented with 100 ng/mL FGF2 and 10 μM forskolin. ② DMEM:HAM F12 (3:1) containing 10% FBS, 1% penicillin/streptomycin supplemented with 20 ng/μL EGF and 40 ng/μL FGF NCC→CEC: ① Dual Smad inhibitor medium (a basal medium supplemented with 500 ng/mL human recombinant Noggin and 10 uM SB431542). ② A basal medium supplemented with 10 µM SB431542 and 3 µM CHIR99021 and a Dual Smad inhibitor medium	Marta et al. (2021)
ASCs	Human	Non-genetic direct reprogramming of recombinant cell-penetrating proteins Oct4/Klf4/Sox2, small molecules (purmorphamine, RG108 and other reprogramming chemical reagents) and biomimetic platforms of simulate microgravity bioreactor were used	Dai et al. (2014)
UC-MSCs	Human	CEC-inducing medium [Eagle’s minimum essential medium supplemented with 0.5 μM BIO (glycogen synthase kinase 3β inhibitor), 10 μM Y-27632, 1% insulin, transferrin, selenium solution, 4 nM triiodothyronine, 0.5 μg/mL hydrocortisone, 50 μg/mL ascorbic acid, 1 mM CaCl_2_, 1 mM sodium pyruvate, MEM amino acid, and MEM essential vitamin mixture]	Yamashita et al. (2018b)
UC-MSCs	Human	CEC differentiation induction medium (human endothelial-SFM supplemented with 0.1% polyvinyl alcohol, 0.5 mM BIO, 10 µM Y-27632, 1% insulin-transferrin-selenium, 0.02 mg/mL 2-phosphate ascorbic acid, 1 μM SB431542, 2.5 μM H-1152 and 0.2 mg/mL CaCl_2_)	Ye et al. (2022)
NCCs	Rat	Differentiated medium (a mixture of conditioned medium collected from cultures rat CEC and DMEM/F12 at a ratio of 3∶1 with 10% FBS)	Ju et al. (2012)
BEPCs	Human	Conditioned medium collected from cultured fetal CEC	Shao et al. (2011)
COPs	Human, mouse	Specific endothelium-inducing medium (Eagle’s minimum essential medium supplemented with 1.0% FBS, 1 μM all-trans retinoic acid, 1 μM BIO, 5 ng/mL TGF-β2, 10 μM Y-27632, 1 μM insulin, 1 mM CaCl_2_, 1 mM sodium pyruvate, 100 U/mL penicillin, 100 μg/mL streptomycin, 1×MEM amino acid, 1×MEM essential vitamin mixture)	Hatou et al. (2013)
Oral mucosa epithelial cells	Rabbit	A mixture of medium collected from cultured feeder cells and basal medium (DMEM/F12, 5∼10% fetal calf serum, 1∼5 μg/mL insulin, 10∼20 μg/mL EGF, 0.5% DMSO and 1% antibiotics)	Jiang et al. (2014)
SKPs	Human	Co-cultured with HCEC-B4G12 cells in human endothelial -SFM supplemented with 10 ng/mL human recombinant bFGF	Shen et al. (2017)
SKPs	Mouse	Corneal Endothelium Inducing Medium [Eagle’s minimum essential medium supplemented with 5% FBS, 1% Insulin, Transferrin, Selenium Solution, 1 mM all‐trans retinoic acid, 0.5 mM BIO, 5 ng/mL TGF-β2, 10 µM Y‐27632, 1 mM insulin, 1 mM CaCl_2_, 1 mM sodium pyruvate, 100 U/mL penicillin Minimum Essential Medium (MEM), 100 mg/mL streptomycin, amino acid and MEM essential vitamin mixture]	Inagaki et al. (2017)

PSCs, pluripotent stem cells; EFSC, eye field stem cells; DMEM/F12, Dulbecco’s modified Eagle medium/Nutrient mixture F-12; BSA, bovine serum albumin; MEM, minimum essential medium; KSR, knockout serum replacement; FBS, fetal bovine serum; P/S, penicillin/streptomycin; NCSC, neural crest stem cells; CEC, corneal endothelial cells; iPSC, induced pluripotent stem cells; MZOC, multi-zone ocular cells; COPs, cornea-derived precursors; NEAA, non-essential amino acids; EGF, epidermal growth factor; NCCs, neural crest cells; ASCs, adipose tissue-derived mesenchymal stem cells; bFGF, basic fibroblast growth factor; SCs, embryonic stem cells; POMPs, periocular mesenchymal precursors; LECs, lens epithelial cells; BEPCs, bone marrow-derived endothelial progenitor cells; UC-MSCs, umbilical cord-derived mesenchymal stem cells; TGF, transforming growth factor; SKPs, skin-derived precursors.

## 6 Strategy of 3D bioprinting for corneal construction

### 6.1 Design requirements for 3D bioprinted corneal grafts

3D bioprinted corneal grafts should have high biocompatibility, mechanical strength and transparency to avoid posttransplant immune rejection and ensure corneal tissue function. Transparency is the key characteristics for ensuring visual function.

High-precision resolution printing technologies should be applied to mimic the microstructure of the natural cornea. During the printing process, appropriate seed cells with the ability to differentiate into specific corneal layers should be selected. In addition, appropriate bioinks need to be developed to support the growth, proliferation, and functional expression of the seed cells.

During corneal epithelial layer construction, cells with high proliferation and differentiation capabilities need to be identified. In corneal stromal layer construction, emphasis should be placed on the arrangement and organization of collagen fibers. On this basis, bioink and seed cell components that support collagen fiber formation need to be applied. The key to corneal endothelial layer construction is the density and monolayer arrangement of seed cells. In addition, the bioink of endothelial products should have strong histocompatibility and be able to support cell survival and functional expression.

### 6.2 3D bioprinting techniques used in cornea construction

Wu et al. first applied 3D bioprinting to corneal tissue engineering in 2016, indicating the potential of this technique for corneal reconstruction (Wu et al., 2016). Since then, several studies in this field have explored the possible applications of various bioprinting techniques (Table 2) (Wu et al., 2016; Isaacson et al., 2018; Kim et al., 2018; Sorkio et al., 2018; Kim et al., 2019a; Zhang et al., 2019b; Duarte et al., 2019; Kilic Bektas and Hasirci, 2019; Park et al., 2019; Kutlehria et al., 2020; Mahdavi et al., 2020; Gibney et al., 2021; Zhong et al., 2021; Mörö et al., 2022; Peng et al., 2022; Tutar et al., 2022; Zhong et al., 2022; Boix-Lemonche et al., 2023; Jia et al., 2023). In this section, the advantages and disadvantages of the most commonly used strategies are reviewed, and their application in corneal tissue construction is discussed.

**TABLE 2:** Summary of original studies on 3D bioprinting of cornea construction.

3D bioprinting techniques	Biomaterials	Cell types	Product	*In vivo* experiments	Main findings	References
Stereolithography	GelMa	Human stromal cells	Stroma	—	The effect of GelMa concentration on corneal stroma reconstruction was investigated	Mahdavi et al. (2020)
Stereolithography	GelMa	rbLSCs	Epithelium	—	A dual ECM model was developed, which can support both the active and reversible quiescent statues of rabbit limbal stem cells	Zhong et al. (2021)
Stereolithography	GelMa, HAGM	hCjSCs, HUVECs, 10T1/2 cells and macrophages	Pterygium	—	The first 3D *in vitro* disease model for pterygium was developed	Zhong et al. (2022)
Stereolithography	Poly-NAGA-GelMa	hCE-Ts, hKs, hUTCECs	Intrastromal lenticule	*In vivo* experiment in rabbits	A functionally effective and bio-safe intrastromal lenticule was developed	Jia et al. (2023)
Stereolithography	PEG, GelMa	rbCECs, rbACSs	Epithelium and stroma	*In vivo* experiment in rabbits	An epithelium/stroma bilayer hydrogel implant	He et al. (2022)
Stereolithography	dECM, GelMa	human corneal fibroblasts	Stroma	*In vivo* experiment in rabbits	A novel bioink of GelMa and dECM mixture was proposed	Zhang et al. (2023)
Inkjet bioprinting	Collagen, agarose	CSKs	Stroma	—	A strategy for corneal stroma construction was proposed	Duarte et al. (2019)
Extrusion-based bioprinting	Collagen, gelatin, alginate	Human corneal epithelial cells	Epithelium	—	The first use of 3D bioprinting in corneal epithelium construction	Wu et al. (2016)
Extrusion-based bioprinting	Collagen, alginate	CSKs	Stroma	—	The first use of 3D bioprinting in corneal stroma construction	Isaacson et al. (2018)
Extrusion-based bioprinting	Gelatin	Ribonuclease 5-overexpressing human corneal endothelial cells	Endothelium	*In vivo* experiment in rabbits	The first use of 3D bioprinting in corneal endothelium construction	Kim et al. (2018)
Extrusion-based bioprinting	GelMa	CSKs	Stroma	—	A strategy for corneal stroma construction with adequate mechanical strength was developed	Kilic Bektas and Hasirci (2019)
Extrusion-based bioprinting	dECM	Keratocytes (differentiated from hTMSCs)	Stroma	*In vivo* experiment in rabbits	A strategy using extrusion-based bioprinting to simulate the microstructure of the collagen arrangement in natural corneal was developed	Kim et al. (2019a)
Extrusion-based bioprinting	dECM	Keratocytes (differentiated from hTMSCs)	Stroma	*In vivo* experiment in rabbits	Swept-source optical coherence tomography test was proposed to be a promising procedure to monitor the biocompatibility of 3D bioprinting construction	Park et al. (2019)
Extrusion-based bioprinting	Alginate, gelatin	Human corneal epithelial cells	Epithelium	—	A strategy combining digital light processing and extrusion-based bioprinting for corneal construction was developed	Zhang et al. (2019b)
Extrusion-based bioprinting	Alginate, gelatin	CSKs	Stroma	—	A strategy for rapid corneal construction combining stereolithography, extrusion-based bioprinting and micro-transfer molding techniques was developed	Kutlehria et al. (2020)
Extrusion-based bioprinting	HA-DA-CDH, HA-CDH, HA-Ald	CSKs (differentiated from hASCs) and neurons (differentiated from hPSC)	Stroma	—	A neuron combined corneal stroma was constructed	Mörö et al. (2022)
Extrusion-based bioprinting	Collagen, alginate, TTC	ASC, BMSC and CS-MSC	Stroma	—	A 3D bioprinting cellularized hydrogel was developed for corneal regeneration	Boix-Lemonche et al. (2023)
Extrusion-based bioprinting	dECM	hTMSCs	Stroma	—	A bioink with the ability to induce stem cell differentiation	Kim et al. (2019b)
Extrusion-based bioprinting	HA-ALD, HA-DA-CDH	hASCs	Stroma	—	A novel strategy for multi-material extrusion-based bioprinting was proposed	Puistola et al. (2024)
Laser-assisted bioprinting	Collagen, recombinant human laminin-521	hESC-LESC, hASC	Epithelium and stroma	—	A layered epithelial and stromal construction was developed	Sorkio et al. (2018)

GelMa, gelatin methacryloyl; rbLSCs, rabbit limbal stem cells; ECM, extracellular matrix; HAGM, hyaluronic acid glycidyl methacrylate; hCjSCs, human conjunctival stem cells; HUVECs, human umbilical vein endothelial cells; NAGA, N-Acryloyl glycinamide; PEG, Poly (ethylene glycol); hCE-Ts, human corneal epithelial cells transfected with SV40; hKs, human keratocytes; hUTCECs, human umbilical cord mesenchymal stem cells transdifferentiated corneal endothelial cells; CSK, corneal stromal keratocytes; dECM, decellularized extracellular matrix; hTMSCs, human turbinate derived mesenchymal stem cells; HA-DA-CDH, carbodihydrazide conjugated dopamine-modified hyaluronic acid; HA-CDH, carbodihydrazide-modified hyaluronic acid; HA-Ald, Aldehyde-modified hyaluronic acid; ASCs, adipose tissue-derived mesenchymal stromal cells; BMSCs, bone marrow-derived mesenchymal stromal cells; CS-MSCs, corneal stroma-derived mesenchymal stromal cells; hESC-LESCs, human embryonic stem cell-derived limbal epithelial stem cells; hASCs, human adipose tissue-derived stem cells.

#### 6.2.1 Extrusion-based bioprinting

Extrusion-based printing can utilize high-viscosity bioinks with high concentrations of cells. The corneal stroma requires high mechanical strength to maintain the shape of the cornea and protect the intraocular tissue. In addition, the functions of corneal endothelial cells and epithelial cells require the formation of intercellular junctions and therefore a higher cell density in the bioink. Therefore, extrusion printing is a suitable 3D bioprinting strategy for corneal tissue construction.

A corneal epithelial system consisting of human corneal epithelial cells, collagen, gelatin and alginate with a controllable degradation rate was constructed using extrusion bioprinting by Wu, which was a milestone in the beginning of 3D bioprinting in corneal tissue engineering (Wu et al., 2016). Since then, various corneal epithelial, stromal and endothelial layers have been constructed using a similar strategy, and high cell viabilities (80∼98%) were found for each of these products (Wu et al., 2016; Isaacson et al., 2018; Kim et al., 2018; Kim et al., 2019a; Zhang et al., 2019b; Kilic Bektas and Hasirci, 2019; Park et al., 2019; Kutlehria et al., 2020; Mörö et al., 2022; Boix-Lemonche et al., 2023). Puistola et al. (2024) constructed stromal tissue by printing acellular stiffer bioink and human adipose tissue-derived stem cell-laden softer bioink in alternating filaments and then printing the filaments perpendicularly in alternating layers. The seed cells were observed to grow along the stiff filaments and form a network-like structure, which is similar to the arrangement of stomal cells in natural tissue (Puistola et al., 2024). Interestingly, by applying shear stress induction to control the orientation of the collagen fibrils during the basal printing process, Kim, H. generated a lattice pattern similar to that of the natural cornea (Kim et al., 2019a). The tissue exhibited extremely high biocompatibility in *in vivo* experiments (Kim et al., 2019a).

These studies suggest that the extrusion bioprinting strategy may have potential for the controlled printing of high-resolution microstructures of the cornea, with its ease of operation while maintaining cell viability.

#### 6.2.2 Stereolithography

During stereolithography, high cell viability can be achieved due to the absence of shear stress. However, cellular damage may occur due to free radicals that dissociate from photoinitiators during the crosslinking process (Huh et al., 2021). On the other hand, low concentrations of photoinitiators may affect the mechanical properties and resolution of the structure. Due to its cross-linking mechanism, the overall printing time is sometimes longer than that of other techniques, and the selection of materials with both biocompatible and photocrosslinking properties is currently limited (Xu et al., 2022). Therefore, it is important to find a suitable bioink to maximize the cytoprotective advantage of stereolithography.

Due to its high-resolution printing capability (25–50 μm), this technology has the ability to be applied to the fabrication of transparent and fine structures, making it suitable for corneal tissue engineering (Raman et al., 2016; Zhang et al., 2019b; Heinrich et al., 2019; Kutlehria et al., 2020; Zhong et al., 2021; Zhong et al., 2022; Jia et al., 2023). In 2020, Mahdavi et al. (2020) first printed corneal stroma tissue with human stromal cells using stereolithography appearance (SLA) technology. By taking advantage of the subtle structure of digital light processing (DLP) products, researchers have successfully printed corneal epithelium using human corneal epithelial cells with customized parameters, making it possible to achieve customized printing of personalized high-resolution stromal lenticules (Zhang et al., 2019b). By combining stereo-photic printing with extrusion-based-printing, Kutlehria et al. (2020) printed a multi-scaffold corneal model using SLA to achieve high-throughput bioprinting of corneal stroma with corneal stromal keratocytes. Furthermore, Zhong et al. (2021) printed corneal limbal stem cells using DLP. In 2022, the same team used DLP technology to print different cell layers, and successfully constructed an *in vitro* pathological model of pterygium by coculturing different layers (Zhong et al., 2022). In the same year, He printed an epithelium-stroma bilayer corneal implant, which proved the great potential of DLP in complex tissue construction (He et al., 2022).

#### 6.2.3 Inkjet bioprinting

Inkjet bioprinting is simple and inexpensive, and can maintain high cell viability (80∼90%) (Heinrich et al., 2019). However, there are limitations restricting the use of bioinks with higher viscosity and higher concentrations of cells.

Using inkjet bioprinting, Duarte et al. (2019) constructed corneal stromal tissue. To ensure the viability of corneal stromal cells, a drop-on-demand strategy was applied (Duarte et al., 2019). The product showed natural transparency, and the cells were found to have a morphology similar to that of natural stromal cells (Duarte et al., 2019). However, the mechanical strength of this product was much lower than that of natural corneal tissue, and the printing process took too long (Duarte et al., 2019). Therefore, considerable future research is needed before this product can be used in clinical applications.

#### 6.2.4 Laser-assisted bioprinting

During laser-assisted bioprinting, cells in the bioink are not directly exposed to shear stress; therefore, high cell viability is ensured (Hopp et al., 2005; Mandrycky et al., 2016). Similar to inkjet bioprinting, bioinks must have a certain fluidity, which limits the viscosity and cell concentration of the bioinks (Mandrycky et al., 2016). In addition, the method is not widely used due to the high cost of its components. Therefore, studies on bioprinting parameters are still lacking.

Sorkio et al. (2018) reported the only use of nozzle-free laser-assisted bioprinting in corneal construction. They successfully constructed a layered epithelium and stroma tissue using human embryonic stem cell-derived limbal epithelial stem cells (hESC-LESCs) and hASCs, demonstrating the potential of the strategy in corneal tissue engineering (Sorkio et al., 2018).

### 6.3 Bioinks used in corneal construction

Bioink refers to the mixture of biomaterials and living cells used in 3D bioprinting (Groll et al., 2018). During or after the bioprinting process, bioinks can be stabilized or cross-linked autonomously or under certain conditions to form a 3D structure. An ideal bioink should have appropriate rheological, mechanical and biological properties to ensure the adaptation of the patient and the function of the printed tissue. In this section, an overview of the current usage of bioinks in corneal tissue engineering will be provided, and their applicability will be discussed.

Hydrogels are a class of biomaterials commonly used in tissue scaffold construction (Gungor-Ozkerim et al., 2018). As a type of alternative bioink, hydrogels are widely used in the 3D bioprinting of corneal tissues. The commonly used hydrogels in corneal engineering include collagen, gelatin, methacryloyl gelatin (GelMa), alginate, hyaluronic acid (HA) and decellularized extracellular matrix (dECM). Collagen, gelatin, alginate, HA, and dECM are natural hydrogels with excellent biocompatibility and low immunogenicitythat can enhance cell survival and reduce the risk of adverse reactions (Ashammakhi et al., 2019; Cui et al., 2020). GelMa is a semisynthetic polymerthat can undergo light-induced cross-linking while retaining biocompatibility, improving the mechanical strength and stiffness of the material, and is suitable for the construction of corneal stroma tissue (Chi et al., 2023).

#### 6.3.1 Collagen

Collagen is the main structural protein that makes up the corneal stroma ECM. It has excellent biocompatibility and can improve the function of printed cells in the product (Sorushanova et al., 2019). Therefore, collagen is widely used in corneal stroma bioprinting (Isaacson et al., 2018; Sorkio et al., 2018; Duarte et al., 2019; Boix-Lemonche et al., 2023). However, collagen itself lacks the mechanical strength to maintain the stability of the corneal stroma and collagen-based materials require further stabilization steps (Alaminos et al., 2006). Therefore, Duarte et al. added agarose to the bioink during the drop-on demand process to improve the stability of the corneal stromal product (Duarte et al., 2019). However, the mechanical strength of the bioink at 20% strain (18.1 ± 3.5 kPa) was still lower than the stiffness of natural corneal tissue (∼300 kPa), so the composition of the bioink still needs further adjustment when used for corneal stroma printing (Garcia-Porta et al., 2014; Duarte et al., 2019). However, the bioink might be a possible choice for bioprinting corneal epithelial or endothelial grafts, which do not require as much mechanical strength as the stroma. Using a bioink containing collagen I and recombinant human laminin, which are components that are similar to natural ECM, Sorkio successfully printed a cell-free layer superimposed on the hASC layer to mimic the corneal stromal structure (Sorkio et al., 2018). However, similar to the bioinks used to make Duarte’s product, Sorkio’s bioinks had to be printed on a nontransparent matriderm to stabilize the structure (Sorkio et al., 2018). Therefore, when using collagen-based bioink to print stromal structures, it is necessary to find suitable components to optimize the stability of the product.

#### 6.3.2 Gelatin

Gelatin is a denatured product of collagen formed by hydrolysis of the triple-helix structure of collagen (Zhu and Marchant, 2011). Compared with collagen, gelatin is more soluble, less antigenic, and can cross-link to form hydrogels at lower temperatures (Maurer, 1954; Yue et al., 2015). Kim et al. (2018) produced a corneal endothelium graft using a bioink containing ribonuclease 5-overexpressing human corneal endothelial cells and gelatin on a lyophilized amniotic membrane. *In vivo* experiments in rabbits confirmed that the endothelial graft exhibited functional behavior and relieved corneal stromal edema (Kim et al., 2018). Kim’s study was the first to produce a transplantable endothelial graft using 3D bioprinting technology. The surprising biocompatibility of the gelation was demonstrated. The mechanical properties of gelatin are too poor to be suitable for the construction of corneal stroma tissue. However, due to its excellent biocompatibility, it may have potential for use in the construction of corneal epithelial and endothelial tissues.

#### 6.3.3 Alginate

Alginate is a polysaccharide derived from brown algae (Zhang et al., 2020). This material is widely used in tissue engineering due to its advantages of easy access, nontoxicity and biocompatibility (Hernández-González et al., 2020; Zhang et al., 2020). However, alginate 3D-printed products were found to have poor cell adhesion. In addition, due to the non-biodegradability of alginate, cell proliferation and differentiation are limited in alginate 3D bioprinting products (BJJoMC, 2008; Pati et al., 2014; Wu et al., 2016; Isaacson et al., 2018; Zhang et al., 2019b; Kutlehria et al., 2020). Recently, an alginate/transparent tunicate cellulose nanofibril bioink coated with different types of mesenchymal stem cells was used to print corneal stromal regeneration products (Boix-Lemonche et al., 2023). However, no cells were found in the products after 3 weeks of observation, which reflected the poor biocompatibility of alginate (Boix-Lemonche et al., 2023). Therefore, alginate may not be suitable for stem cell printing.

#### 6.3.4 Hyaluronic acid

HA is a nonsulfated glycosaminoglycan present in the natural ECM that is suitable for bioprinting applications because of its various rheological properties and viscosity (Gungor-Ozkerim et al., 2018). Chemical modification can improve the cytocompatibility and mechanical properties of these materials, expanding their 3D bioprinting applications (Hintze et al., 2022). Wang et al. used HA mixed with GelMa bioink in the bioprinting of rabbit corneal stromal cells (Wang et al., 2022). However, the material requires photocrosslinking in the subsequent processing steps due to the presence of GelMa, which limits its biological function. However, an HA-based bioink was developed by Mörö et al. (2022). In their study, hASCs, hASC-derived corneal stromal keratocytes (hASC-CSK) and human pluripotent stem cell-derived neurons were encapsulated. Compared with carbonated dihydrazide or aldehyde-coupled HA bioink, dopamine-coupled HA bioink exhibited excellent printability, shape fidelity, and optical clarity (Mörö et al., 2022). The dopamine-coupled HA bioink was also reported to show similar mechanical properties to those of photocrosslinked hydrogels even without photocrosslinking, preventing free radical damage from the cross-linking process (Mörö et al., 2022). In addition, due to the presence of dopamine, a neurotransmitter that benefits neuronal growth, the growth of nerve cell axons was detected after 7 days of culture in the product (Mörö et al., 2022). Then, the team extruded printed corneal stroma with hASCs using the same biomaterial and implanted it within the porcine corneal stroma (Puistola et al., 2024). Cell proliferation and keratocyte-like activities were observed within 14 days of follow-up, which indicated the excellent biocompatibility of the bioink (Mörö et al., 2022; Puistola et al., 2024).

#### 6.3.5 Decellularized extracellular matrix

Decellularized extracellular matrix is a material that can be obtained by a variety of decellularization techniques for the preparation of organ engineering scaffolds (Arenas-Herrera et al., 2013). Its leading advantage is its similarity to the composition of natural tissues. Kim et al. evaluated the effect of cornea-derived decellularized extracellular matrix (Co-dECM) as a bioink and found that the material had favorable biocompatibility, which contributed to the maintenance of keratocyte-specific characteristics (Kim et al., 2019b). The team then printed human turbinate-derived mesenchymal stem cells (hTMSCs) encapsulated with Co-dECM and found that hTMSCs derived into corneal stromal cells and performed their functions in an environment mimicking natural components of the ECM (Kim et al., 2019b). Zhang also found that the CECM(dECM)-GelMa bioink could maintain the characteristics of human corneal fibroblasts in the product, leading to the production of a behavior similar to that of resting keratocytes (Zhang et al., 2023). Thus, bioinks containing dECM have the potential to maintain the seed cell phenotype.

However, dECM may be antigenic and carry the risk of transmitting underlying diseases (Kasimir et al., 2006). Therefore, the safety of its applications still needs long-term follow-up studies.

#### 6.3.6 Methacryloyl gelatin

GelMa is a gelatin derivative that is modified with a photopolymerizable methacryloyl group. It can form mild covalent crosslinking after photoinitiated radical polymerization in the presence of ultraviolet (UV) radiation and photoinitiators (Yue et al., 2015; Gungor-Ozkerim et al., 2018). GelMa is a promising bioink that has been widely used in stereolithography (Mahdavi et al., 2020; Zhong et al., 2021; Zhong et al., 2022; Jia et al., 2023). The effect of the GelMa concentration on cell behavior during bioprinting has attracted increasing interest.

The key to the use of GelMa in the construction of corneal tissue is to balance its mechanical stiffness and biocompatibility, and ensure the biological function of the product while maintaining its structural stability. Kilic first used 15% GelMa to encapsulate keratocytes for 3D printing of corneal stroma (Kilic Bektas and Hasirci, 2019). Light curing was used after printing to stabilize the system. Ninety-five percent of the cells were found to survive in the product for 3 weeks (Kilic Bektas and Hasirci, 2019). However, most of the printed cells exhibited a round shape and failed to form cell‒cell communication processes, suggesting that the keratocytes did not perform their natural function in the product. One possible reason for this finding is that the tight network formed by GelMa cross-links restricts the movement and pseudopod extension of keratocytes. Decreasing the GelMa concentration has been reported to ameliorate this situation (Yin et al., 2018). Therefore, Mahdavi et al. (2020) used a lower concentration of GelMa to print corneal stromal cells. Bioinks with 12.5% GelMa were found to be similar to natural stroma in terms of their water content and optical transmittance and exhibited greater mechanical stiffness than bioinks with 7.5% GelMa (Mahdavi et al., 2020). Interestingly, cell compatibility was higher in 12.5% GelMa. The cells were elongated after 7 days of culture, with COL I and lumican formation in the material after 28 days (Mahdavi et al., 2020). With further understanding of the concentration parameters, many promising products have been fabricated by stereolithography using comparatively low concentrations of GelMa, including the first *in vitro* 3D-printed pterygium model and a dual ECM “yin-yang” stromal structure (Zhong et al., 2021; Zhong et al., 2022). To further enhance the mechanical strength of GelMa at low concentrations, Peng et al. added 25% N-Acryloyl glycinamide (NAGA) to 5% GelMa to print a corneal intrastromal lenticule (Jia et al., 2023). The hydrogel network balances the structural stability of the product with biocompatibility while ensuring optical clarity and nutrient permeabilization (Jia et al., 2023). Instead of trying to reduce GelMa concentration, He et al. toughened high concentration GelMa material by adding long-chain poly (ethylene glycol) diacrylate to reduce its intrinsic brittleness (He et al., 2022). The epithelial/stromal hydrogel corneal graft was successfully prepared and showed regenerative potential under the synergistic effect of the orthogonal fiber structure and the encapsulated stem cells (He et al., 2022). Zhang also successfully improved the biocompatibility of the materials by incorporating dECM into GelMa hydrogels (Zhang et al., 2023).

In conclusion, as an important ink in stereolithography printing, the combined use of GelMa with other bioinks may be an important approach to balance its technical advantages and material disadvantages.

### 6.4 Stem cells used in cornea construction

Stem cells were first used for corneal tissue construction in 2018 (Sorkio et al., 2018). At present, the strategies for applying stem cells in 3D bioprinting mainly include printing stem cells directly or adding stem cell-derived corneal cells to bioink. The selection and differentiation of stem cells are the basis of seed cell culture. During the printing process, the survival of stem cells and the interaction between stem cells and bioink should be emphasized. The differentiation, proliferation and integration of stem cells in corneal tissue during follow-up observations determine the visual and mechanical function of the printed product. Stem cells have been used for 3D bioprinting of corneal epithelium and corneal stroma constructs. However, the application of stem cells for corneal endothelial tissue needs to be explore urgently. In this section, the research progress on the application of stem cells in 3D bioprinting of corneal tissue will be summarized.

#### 6.4.1 Applications of stem cells in corneal epithelial layer construction

Primary LSCs or LSCs derived from stem cells are mainly used for the construction of corneal epithelial tissue. Sorkio et al. printed 3-layer stacked cell layers by laser-assisted bioprinting using hESC-derived LESCs mixed with human recombinant laminin-521, which was the first 3D bioprinted corneal tissue using stem cells (Sorkio et al., 2018). After 7 days of printing, the cells showed significant proliferation and expressed the corneal progenitor cell markers p63α and p40 (Sorkio et al., 2018). After 12 days of printing, the proliferation of hESC-LESCs decreased, and CK3, which is a terminal differentiation marker of corneal epithelial cells, was detected in the product (Sorkio et al., 2018). Interestingly, the printed product retained 4 cell layers and exhibited an epithelial tissue-like structure similar to that of the natural cornea (Sorkio et al., 2018).

Another interesting product is a yin–yang scaffold model that encapsulated both active and quiescent LSCs (Zhong et al., 2021). In this study, GelMa and hyaluronic acid glycidyl methacrylate (HAGM) were used (Zhong et al., 2021). Both materials were able to support LSC viability, but the cells in the GelMa group expressed Ki67 and were proliferative, whereas the cells in the HAGM group did not express Ki67 and presented a reversible quiescent state (Zhong et al., 2021). Although this work does not focus on the construction of corneal tissue, it provides an effective 3D model for the *in vitro* study of LSC proliferation state transition (Zhong et al., 2021).

Both studies focused on the viability, proliferation and marker protein expression of stem cells in 3D-printed products. However, further *in vivo* animal experiments need to be completed to determine the function of 3D-printed epithelial tissues under physiological conditions.

#### 6.4.2 Applications of stem cells in corneal stromal layer construction

The stem cells used for 3D bioprinting of corneal stroma are mainly MSCs. Researchers have explored the effects of printing ASCs and TMSCs. Notably, ASCs have been widely explored in corneal stromal 3D bioprinting due to their easy availability and ability to differentiate into mature cells.

Stem cells can be encapsulated in bioink before or after the induction of differentiation. The timing of stem cell encapsulation may affect the cell density and function of the cells in the product. Mörö et al. found that compared with hASCs-CSK, hASCs had a greater survival rate during the printing process (Mörö et al., 2022). The differentiation induction of hASCs after printing was carried out, and it was found that both the functional proteins and intercellular junctions of hASCs with or without differentiation induction after printing were increased (Mörö et al., 2022). However, compared with those printed with hASCs-CSK, the tissues printed with hASCs showed slight opacities in postprinting observations, which may be related to the strong proliferative capacity of hASCs (Sorkio et al., 2018; Mörö et al., 2022; Puistola et al., 2024). The proliferative capacity of hASCs gradually decreases after the initiation of differentiation (Mörö et al., 2022). Further studies showed that hASC-CSK could enhance tissue integration and induce neuron axon growth into the printed tissue (Mörö et al., 2022). Therefore, the encapsulation of stem cells during printing and differentiation induction after printing may be a better option than printing keratocytes induced from stem cells in advance.

The use of ASCs in 3D bioprinting for corneal stroma tissue has been found to have the following advantages. First, ASCs can differentiate into corneal stromal keratocyte-like cells with elongated cell morphology and cell networks (Mörö et al., 2022). These cells were found to express keratocan, ALDH3A1, aquaporin and more lumican (He et al., 2022). Lumican is considered beneficial for preventing fibroblast activation and corneal scar formation (He et al., 2022). Second, ASCs can enhance the mechanical strength of the product and reduce the swelling rate of the product (Puistola et al., 2024). Moreover, as mentioned earlier, ASCs may enhance product integration and promote surgical incision healing. A study conducted on *ex vivo* porcine eyes showed that 3D bioprinted stromal constructs displayed interaction and attachment to host tissues in corneal organ cultures (Sorkio et al., 2018). In addition, Puistola et al. (2024) reported that ASCs promoted the outgrowth of nerve axons into grafts, which may contribute to the repair of corneal tissue through neurotrophic effects.

## 7 Conclusion and prospects

To alleviate the shortage of donor corneal grafts, alternatives need to be developed urgently. As an emerging technology, 3D bioprinting has promising applications in corneal tissue engineering. First, suitable bioinks need to be identified (Wu et al., 2016; Isaacson et al., 2018; Kim et al., 2018; Sorkio et al., 2018; Zhang et al., 2019b; Mahdavi et al., 2020). Subsequently, a variety of bioprinting strategies constructing structured corneal tissue have been attempted (Sorkio et al., 2018; Kim et al., 2019a; Duarte et al., 2019; Kilic Bektas and Hasirci, 2019; Zhong et al., 2022). However, the demand for lamellar keratoplasty is increasing in clinical practice. Therefore, more attention should be given to the construction of corneal stromal and endothelial tissue. Corneal cells play important roles in maintaining the transparency and barrier function of the cornea. However, due to the difficulty in expanding primary cells *in vitro* and the shortage of donor sources, it is important to find suitable seed cells for the construction of functional corneal tissue grafts. Seed cells should be proliferation-controllable, safe, and resistant to the bioprinting process. Stem cells have strong proliferation and differentiation capabilities, making them suitable as seed cells for large-scale production of corneal grafts. Recent works on the application of stem cells in 3D bioprinting of the cornea have focused mainly on the construction of corneal stroma tissue. Initial attempts used cells derived from stem cell differentiation for printing. However, with a better understanding of the behavior of stem cells in bioprinting products, the use of undifferentiated stem cells as seed cells in bioprinting products has gradually increased.

In addition, a variety of biomaterials and printing strategies have been used for corneal tissue construction, and the function and behavior of seed cells in the printed products have been observed. Due to the impact of biomaterials and printing strategies on the vitality and functions of seed cells, appropriate bioinks and printing methods should be selected for stem cell applications. Interestingly, the effect of matrix stiffness on corneal cell function may provide the possibility of applying 4D bioprinting for corneal regeneration.

The epithelial layer of the corneal graft is not usually necessary for keratoplasty. However, it is necessary to provide a basement membrane-like anterior surface for corneal epithelial cells to attach.

The products of the corneal stromal layer require high transparency, structural accuracy, and mechanical strength. Therefore, it is better to choose biomaterials and printing strategies with corresponding properties and add cells to the bioink to enhance its tissue integration. HASCs have shown great promise in the construction of corneal stroma, but the transparency of the printed materials still needs to be further improved. A recent study proposed a strategy to print smooth corneal structures on the order of microns, which is highly important for the customized printing of corneal grafts (Zhang et al., 2024).

The 3D bioprinting of corneal endothelial grafts remains to be studied. Unlike the epithelium and stroma, the corneal endothelium is a single layer of cells and does not require the construction of complex three-dimensional structures. However, the mechanical strength, morphological curvature and biocompatibility of corneal endothelial grafts greatly affect intraoperative implantation, postoperative graft adherence and corneal endothelial cell survival. Although the stem cell source of corneal endothelial cells has been extensively studied, the application of stem cells in 3D bioprinting of artificial corneal endothelium is still unexplored. The therapeutic effect of cell injection for corneal endothelial diseases has been gradually demonstrated (Wong and Mehta, 2022). However, the construction of commercialized corneal endothelial grafts is highly important for full-thickness corneal tissue engineering and for ophthalmology hospitals without cell laboratories.

In conclusion, the application of stem cells in 3D-printed corneal tissue engineering has promising prospects. However, criteria to ensure the safety of stem cells should be established. In addition, the source of stem cells still faces ethical challenges. Commercial and clinical trials should be carried out with caution until the standards for the use of stem cells are improved. Future research will focus on high-throughput manufacturing and safety observation of 3D bioprinted grafts to alleviate the shortage of corneal donor grafts.
